# P02-023 - NLRP3 mosaicism as a cause of late-onset CAPS

**DOI:** 10.1186/1546-0096-11-S1-A130

**Published:** 2013-11-08

**Authors:** Q Zhou, AK Ombrello, D Chin, DL Kastner, I Aksentijevich

**Affiliations:** 1 Inflammatory Disease Section, National Human Genome Research Institute (NHGRI/NIH), Bethesda, USA

## Introduction

The dominantly inherited cryopyrin-associated periodic syndromes (CAPS) are caused by heterozygous missense gain-of-function mutations in the *NLRP3* (*CIAS1*) gene encoding NLRP3 (also known as cryopyrin). Most patients present at a young age with a variety of clinical symptoms including fevers, urticaria-like skin rash, arthropathy, and CNS inflammation. A subset of patients followed at the National Institute of Health’s autoinflammatory disease clinic has adult-onset fevers and urticarial rash but conventional genetic testing has been unremarkable for any mutations in *NLRP3*. We analyzed one such "mutation-negative" patient. She is a 63y/o female of Irish ancestry who developed a gradually worsening stress-induced urticarial rash in her 40s. Additional clinical history is remarkable for severe arthralgia, myalgia, chills, and occasional conjunctivitis. Initially started on anakinra 100mg/day in 2003, she had dramatic improvement in symptoms; however, her anakinra dose has required periodic adjustments since that time to control her symptoms.

## Objectives

To identify the cause of disease in a patient who presented with late-onset CAPS and who was mutation-negative in *NLRP3* based on the conventional Sanger sequencing.

## Methods

We performed whole exome sequencing in the DNA sample extracted from peripheral blood. A total of 192 subclones were randomly selected and subjected to Sanger sequencing to validate the *NLRP3* somatic mutation. Targeted deep sequencing on *NLRP3* will be done using the DNA from blood and buccal cells.

## Results

Our established exome data analysis pipeline failed to identify any plausible candidate gene in this patient. In order to increase the sensitivity of variant calling, we used a different combination of parameters including calling SNPs/Indels with extreme strand bias, which resulted in a rapid increase in false positive rate but also raised the possibility of identifying somatic mosaicism mutations. Based on this new analytic approach, we identified a possible mutation p.Tyr570Cys (c.1709A>G) in *NLRP3*. Exome data showed that there are 12 reads carrying the mutant G allele and 64 reads carrying the wild type A allele (15%). This mutation has been previously reported in a NOMID/CINCA patient from Australia. The putative mosaic p.Tyr570Cys mutation was validated by subcloning a 718bp fragment from the patient’s leukocyte DNA and subsequent Sanger sequencing of 192 clones. We found 20 clones carrying the p.Tyr570Cys mutation, which established the level of somatic mosaicism at 10.4%. We are in the process of evaluating this mosaic mutation in other tissues from this patient.

## Conclusion

We report *NLRP3* somatic mosaicism in a patient presenting with a late onset CAPS. To our knowledge this is the first time that a *NLRP3* mutation has been identified in patients developing symptoms in adulthood. Other possible causal genes have been ruled out based on exome sequencing analysis. Our result contributes significantly to understanding the pathogenesis of disease in patients with adult-onset autoinflammatory diseases.

## Disclosure of interest

None declared.

